# Parents providing palliative care for children with cancer

**DOI:** 10.3332/ecancer.2024.1724

**Published:** 2024-07-01

**Authors:** Rima Saad Rassam, Huda Abu-Saad Huijer, Samar Noureddine, Ellen M Lavoie Smith, Joanne Wolfe, Souha Fares, Miguel R Abboud

**Affiliations:** 1 Hariri School of Nursing, American University of Beirut, Beirut 1107 2020, Lebanon; 2 Department of Pediatric Medicine, St Jude Children’s Research Hospital, Memphis, TN 38105, USA; 3 Faculty of Health Sciences, University of Balamand, Al-Kurah, Lebanon; 4 School of Nursing, University of Alabama at Birmingham, Birmingham, AL 35294, USA; 5 Department of Pediatrics, Mass General Hospital for Children and Harvard Medical School, Boston, MA 02114, USA; 6 Department of Pediatrics and Adolescent Medicine, American University of Beirut Medical Center, Beirut 1107 2020, Lebanon; a https://orcid.org/0000-0002-8232-8733; b https://orcid.org/0000-0001-6959-9419; c https://orcid.org/0000-0001-5133-0913; d https://orcid.org/0000-0002-6519-6636; e https://orcid.org/0000-0002-4406-7413; f https://orcid.org/0000-0001-8547-2565; g https://orcid.org/0000-0001-8469-6823

**Keywords:** paediatric, oncology, palliative care, parents

## Abstract

Parents of children with cancer provide paediatric palliative care (PPC). However, the activities they perform remain underexplored, especially in low- and middle-income countries (LMICs) where the care heavily relies on family involvement. The aim of this study is to identify parental PPC tasks and intentions to perform PPC tasks and to determine their associated factors. A quantitative cross-sectional descriptive design was used to recruit parents of children with cancer from three major paediatric oncology centres in Lebanon. Data were collected through structured interviews using an adapted questionnaire. The statistical analyses included descriptive, bivariate and regression analyses of PPC tasks and intentions. One hundred and five participants completed the study. On average, parents performed 22 PPC activities. The findings suggested statistically significant associations of the number of PPC tasks with the participants’ marital status, number of people living with the child, the intentions to perform the tasks and the number of the child’s symptoms in the previous week. Examining parents’ tasks in PPC in LMICs, such as Lebanon, enhances knowledge of PPC practice in these regions and informs improvement strategies. These results promote PPC understanding, highlight factors influencing PPC delivery and provide a useful measure of PPC tasks performed by parents of children with cancer.

## Introduction

Over the last two decades, paediatric palliative care (PPC) has been a growing field within the paediatric oncology specialty [1]. According to the World Health Organisation (WHO), PPC is comprehensive care that aims to alleviate the suffering of children with serious illness and their families, and it involves a multidisciplinary approach to supporting the family [2]. Sustainable access to this approach requires its integration within all healthcare levels, including community health centres and children’s homes [2]. In 2014, the World Health Assembly issued a global resolution for palliative care as part of universal health coverage to promote accessibility to palliative care as a ‘human right’. The resolution particularly highlighted the need to train healthcare professionals and family members to reduce patient suffering [3]. Globally, a Lancet Commission study reported that 98% of children who die from serious health-related suffering from diseases (such cancer and other conditions) reside in low- and middle-income countries (LMICs), accentuating the need for PPC in these regions [4].

In high-income countries (HICs), specialised PPC services are often available and well-established [5, 6]. Several paediatric oncology professional organisations have adopted the WHO definition to establish principles and standards for PPC provision [7–9]. In LMICs, the lack of resources hinders PPC development and provision [10, 11]. Nevertheless, several PPC programs in hospitals or hospices in LMICs exist primarily in urban zones [12]. Alternatively, home-based services are provided to balance accessibility, continuity of care and symptom management through coordination between healthcare providers and the family [12, 13]. The cultural context may also encourage the home setting as a preferred option respecting family bonding [14]. The scarcity of resources and the presence of specific cultural features in LMICs seem to intensify the family role, particularly in PPC delivery.

The literature describing the parents’ role in PPC identifies them as care recipients while recognising them as unique caregiving agencies [15, 16]. As caregivers, parents, especially the primary caregivers, are pillars in providing PPC based on their views, training and skills, especially in LMICs, where the needs are high. Qualitative and quantitative reports have revealed a myriad of caregiving tasks that parents performed during the illness journey [17–23]. The parental PPC tasks for a child with cancer vary in nature and intensity according to the child’s condition [17]. Parental caregiving requires an expanded role to cover medical, technical and emotional aspects related to the child’s condition [18]. In the Netherlands, Verberne *et al* [19] described four clusters of tasks: 1) providing basic and complex direct care ranging from assisting with activities of daily living to manipulating sophisticated medical equipment; 2) coordinating the care with the healthcare team; 3) decision-making, from simple daily judgements to goals of care decisions; and 4) ensuring family balance [19]. In Western New York in the United States, authors identified additional PPC tasks, namely dealing with the challenges of getting necessary equipment and medications, sharing their own experiences with similar caregivers and praying with the child [20]. In complex cases, caregiving activities are intensified. Authors from Italy reported that the time spent on tube feeding, handling respiratory devices and managing pain and seizures reached 9 hours a day [21]. Studies conducted in the United States highlighted decision-making as a major task among parents of patients with terminal illnesses [22, 23].

The above studies emanate from HICs and often address PPC from the narrow angle of end-of-life. To the best of our knowledge, no studies have explored parental PPC tasks throughout the disease trajectory in LMICs. Identifying these tasks and their associated factors is needed to design necessary interventions and training to ensure successful performance and, ultimately, to contribute to enhancing PPC outcomes. Moreover, the exploration of parental PPC tasks informs the potential parental need for education, social or medical support, or respite care. In the context of LMICs, a clear description of how parents provide PPC is essential because of their prominent role in delivering care. This study aims to identify parental PPC tasks and intentions to perform PPC tasks and to determine the factors related to both elements.

## Methods

### Design, sample and setting

The study was conducted by using a quantitative cross-sectional descriptive design. A non-probability convenience sampling design was used to recruit parents of children with cancer who met the following inclusion criteria: speaking Arabic, being Lebanese or officially permanent residents in Lebanon (including Syrian, Iraqi and Palestinian nationalities), and being the primary caregivers of a child with cancer on active cancer treatment for more than 2 months. In this study, we defined primary caregivers as the mother, father or significant other who is the first line of healthcare support for the child. Recruitment occurred in the inpatient and outpatient facilities of three major paediatric oncology centres in Lebanon: The Children’s Cancer Institute at the American University of Beirut Medical Centre, the Saint George Hospital University Medical Centre, and the Lebanese Hospital Geitaoui-University Medical Centre. All three sites receive a large number of Lebanese and Arab children with cancer from various socioeconomic backgrounds. The centres provide state-of-the art cancer therapies for children with various cancer diseases, using a multidisciplinary approach to address the child’s and family’s needs. None of the three centres has a specialised PPC team. One centre has an adult palliative care team that collaborates in PPC services. Therefore, PPC provision in the three centres depended on the paediatric oncology teams and the parents.

### Data collection

The ethical and administrative approvals were secured from the sites before initiating the study. The data collection was conducted between August and November 2021. The first author directly approached parents during the child’s visit to the centre while implementing COVID-19 precautions. Oral consents were obtained from participants before completing the study. Data were collected by the researcher through structured interviews exclusively via a WhatsApp call arranged at a mutually suitable time with consenting participants.

### Study instruments

The study questionnaire included three sections divided into demographic data, clinical data and parental PPC tasks. The items were adapted from previously validated tools and new items were added based on the literature review and the study purpose. The demographic data section solicit information about the parent and the child with cancer. The clinical data section comprised questions about symptom experience adapted from the Arabic version of the Memorial Symptom Assessment Scale (MSAS) [24, 25]. Based on the literature on symptoms in paediatric oncology, the ten most prevalent symptoms were listed in the survey. The MSAS total score ranges between 0 and 4 and represents the mean range and standard deviation of symptom scores (calculated as the average of frequency, severity and distress of each symptom). A higher score indicates a more intense symptom experience [26].

The section on parental PPC tasks used an adapted version of the Care of My Child with Cancer (CMCC) scale after obtaining the author’s permission [27]. The parental PPC tasks section included 31 PPC caregiving tasks performed by the parent. For each task, participants were asked to answer (yes/no) whether they performed the task within the previous week if applicable. For tasks not performed, participants were asked to rate their likelihood of performing the task in the coming week on a five-point Likert scale. The list of PPC tasks was adapted CMCC scale, which includes a physical and an emotional subscale [27]. In several studies, the CMCC demonstrated sound psychometric properties [27–30]. To fit the study context, 22 of 28 tasks were selected from CMCC, and some items were reworded to enhance clarity. The items removed were not within the scope of activities that parents perform within the study context (such as changing central line dressing and flushing or giving Intravenous injection). The answer options were modified to ‘yes/no/not applicable’ instead of ratings of the time required and degree of effort on a five-point Likert scale. Because CCMC was validated among parents of children with cancer excluding terminal phases, it partially covers the PPC context. Therefore, nine items were added based on the literature to examine parental PPC caregiving activities across the different phases of the disease trajectory. The revised CCMC tool included 31 items. PPC tasks were measured through binary responses. A score of one was allocated for each task reported as ‘yes’, and zero was allocated for each task reported as ‘No’ or ‘Not applicable’. A summative score was calculated based on the number of tasks performed by the participant in the previous week and ranged from 0 to 31.

PPC intentions were measured using a five-point Likert scale to report the likelihood of performing PPC tasks that were not performed in the previous week. The total PPC intentions score was calculated by computing the mean of participants’ likelihood to engage in PPC tasks in the coming week.

As the survey was developed in English, we conducted a cross-cultural adaptation process before use [31]. The procedure included forward translation to Arabic, synthesis, back translation, content validation by a panel of 10 experts in paediatric oncology and palliative care and pilot testing with 20 participants who were different from the study sample. The prefinal version was refined based on the experts’ review and the pilot results before use in the main study sample.

### Statistical analysis

Data were analysed using Statistical Product and Service Solutions (SPSS) version 26 [32]. Descriptive and correlation statistics were conducted. Nonparametric tests were used when the parametric tests’ assumptions were unmet. All demographic and clinical variables were tested for association with the PPC tasks and intentions. The associations between both outcomes were also examined. Significant associations were set at two-tailed *p* ≤ 0.05. For PPC tasks, the data were suitable for linear regression analysis. The residuals analysis of the regression model was done to test the assumptions (absence of homoscedasticity and presence of linearity and normality).

## Results

### Sample characteristics

A total of 105 of 110 participants completed the study (response rate = 95.4%). The majority were mothers (*n* = 89, 84.8%), homemakers (*n* = 67, 63.8%) and from the Muslim religion (81%). Families included, as a median, four people (Inter quartile range (IQR) = 3–5) living with the child in the same house. Table 1 illustrates the demographic characteristics of the participants.

As for the participants’ children, more than half were female (*n* = 62, 59%), and their age ranged between 5 and 15 years (median = 7, IQR = 4.5–12 years). Almost half of the children had leukemia (*n* = 52, 50%) that was in remission (*n* = 58, 55%). Most children were receiving disease-directed therapy (*n* = 98, 93%). As reported by the participants, only two children (2%) were receiving palliative treatment. Most parents (*n* = 84, 81%) reported that the chances of cure for their child are either very high (*n* = 53, 51%) or somewhat high (*n* = 32, 31%). The median caregiving duration was 1.5 years (IQR = 0.5–3). Children required, on average, 8.7 hours of care daily (SD = 5.7). More than half of the children (*n* = 62, 59.1%) experienced at least four symptoms in the last week. Table 2 illustrates the symptoms prevalence and scores.

### Pediatric palliative care tasks and intentions

When asked about PPC tasks performed during the last week, on average, participants reported engaging in 22 activities (SD = 2.8) of 31. The mean of intentions to participate in activities not performed over the last week was 2.79 (SD = 0.5) of 5. Table 3 presents the distribution of activities and the intention score when activities were not performed. Participants also added to the list other activities that they performed during the previous week as follows: studying with the child/preparing for school (mentioned by 12 participants), walking in nature (mentioned by two participants), cooking with the child (mentioned by two participants), doing physiotherapy sessions, visiting grandparents and talking with the child about the future (‘dreaming of tomorrow’).

### Factors associated with PPC tasks and intentions

Among the demographic variables, the number of individuals living with the child was positively correlated with PPC tasks (Spearman rho = 0.23, *p* = 0.017). In addition, a Mann-Whitney *U* test revealed that married caregivers performed significantly more PPC tasks in the previous week (median = 23, *n* = 95) than did nonmarried caregivers (median = 19, *n* = 10). These results suggest that family status influences the number of PPC tasks that participants perform. Among the clinical variables, the number of symptoms the child experienced in the previous week was positively associated with the number of PPC tasks (Spearman rho = 0.25, *p* = 0.01). In addition, PPC intention scores were positively and significantly correlated with PPC task scores (Spearman rho = 0.29, *p* = 0.003). When entered in a linear regression model, all four variables predicted the PPC tasks score significantly, explaining 29% of the total variance (*R*² = 0.29, F (4, 100) = 10.196, *p* < 0.001). The findings suggest a significant negative relationship between the caregiver’s age and PPC intentions (Spearman rho = −0.24, *p* = 0.013): the lower the participant’s age, the higher the PPC intentions.

## Discussion

This study is among the first endeavours to highlight the extent of parental involvement in providing PPC for children with cancer, especially in LMICs. Being the first undertaken in Lebanon, the study brings distinctive perspectives from a limited-resource setting. Our findings concur with those in the existing literature, emphasizing the pivotal role of parents in PPC.

As described in previous studies, the PPC tasks in our sample encompass direct physical care and emotional support, managing symptoms, monitoring the patient’s status and making treatment decisions [17, 19, 20, 21, 27, 28, 33]. The high prevalence for meeting the child’s emotional, maintaining the child’s comfort, and obtaining necessary equipment and medications can be attributed to the cultural and contextual aspects of care. These findings highlight the close family bonding between the child and primary caregiver (often the mother) as well as the substantial struggle to secure equipment and medication within a limited-resource setting. As natural caregivers, parents performed, on average, 22 PPC tasks, regardless of prior knowledge or training. Many study participants commented that their engagement in PPC was not conditioned by prior knowledge because they provide ‘PPC by parents’ intuition’ when caring for their sick child. At the same time, participants acknowledged their need for PPC education for better performance, as reflected in the high score on their intention to obtain more information about PPC and discuss PPC with the healthcare team. Many recent studies have described the educational needs among parents of children with cancer [34–36]. These educational needs pertain to the child’s condition; disease and symptom management, including physical and mental care; and communication with the child and the healthcare team. As the child’s comfort is an integral component of palliative care, the involvement of parents in the care becomes natural and instinctive due to their role in comforting the child. Our findings also reflect the prominent role of parents in promoting the spiritual domain of PPC within the Lebanese context. Most participants reported praying with the child (*n* = 88, 84%). Such results align with previous reports describing strong religious commitment in Lebanon within the religious diversity in the country [37, 38]. Taken together, our study results provide more evidence about the concomitant provision of PPC by both parents and professionals [15]. From this standpoint, enhancing caregivers’ training in all the PPC domains promotes their ability to deliver care, thus paving the way to the child’s comfort and better quality of life.

PPC tasks were more prevalent among married caregivers than unmarried ones (single, widowed, separated/divorced). The PPC tasks also increased with the number of persons living with the child. Being married and the number of persons living with the child also predicted PPC tasks as revealed in the regression analysis. These two predictors reflect an evident impact of family life on the child’s care. Most of the caregivers in the sample were females, mothers and married. Being married facilitates the performance of PPC tasks in congruence with the social role of a mother. The small number of participants in other marital status categories (single, widowed, separated/divorced) limited the data analysis for comparison between categories. However, this small number may reflect a reality within Lebanese society, where separation/divorce is still considered taboo and often undisclosed. Likewise, the findings revealed another cultural aspect. Within the Lebanese context, it is common for an extended family to live together and share household tasks. As such, with the increase in the number of persons living with the child, the caregiver may have more time to perform PPC tasks because others can attend to family needs. The two predictors highlight an important cultural aspect of caring for a child with cancer at home in Lebanon.

Our study data also suggested that the number of symptoms predicted PPC tasks. These findings align with previous reports that underscore symptom experience as a fundamental aspect determining PPC needs [39–43]. In the clinical field, the number of symptoms experienced by the child is an important screening criterion for eligibility to PPC. The ‘Paediatric Palliative Screening Scale is one of the screening tools that help ensure timely referrals to specialised PPC services [40]. The scale outlines five domains, among which the ‘symptom and problem burden’ (p.3) has the largest proportion of items, and the number of symptoms is an important attribute within the domain [40]. Our findings reiterated the close link between the child’s clinical status and PPC interventions from the caregivers’ perspectives. Our data did not reveal an association between symptom burden (MSAS score) and PPC tasks, possibly because approximately half of the children had leukemia in remission. However, such a relationship remains a plausible finding to explore in future research.

As for the factors associated with PPC intentions, our findings underscore a negative correlation with the participants’ age. These findings suggest that younger caregivers may be more open to performing PPC tasks than older caregivers are. Although older parents in the study may have more experience in childcare, they may have perceived that PPC tasks require effort and energy that younger parents are more willing to exert. Our sample included relatively young participants (*M* = 37.6 ± 7.6 years). Previous authors reported a higher comprehension of palliative care among younger categories of caregivers [44] and community samples [45, 46]. Moreover, our data highlighted a significant positive association between PPC tasks and intentions. The higher the number of PPC tasks performed in the previous week, the higher the intention to perform other PPC tasks in the coming week. It is possible that parents have learned about PPC tasks through their participation in the study. Thus, they may have expressed their intentions based on acknowledging the importance of their contribution to PPC provision. In this study, the intentions indicate the willingness to perform PPC in the future. Soliciting these intentions sheds light on the future lookout of the participants and their likelihood to engage in PPC tasks. Such understanding informs the design of interventions and training while levelling to the participants’ intentions.

### Limitations

This study filled a knowledge gap regarding parents’ involvement in providing palliative care for children with cancer. However, some limitations may be present. A convenience sample, in addition to having half of the participants’ parents of a child with leukaemia might have weakened the representativeness of the sample, thus limiting the generalisability of the results. Hawthorne effect and interviewer’s subjectivity are possible. However, the researcher emphasised objectivity in addressing the survey items. In addition, some items in the questionnaire were newly developed and not fully validated. The researchers conducted content validation and pilot testing before initiating the study.

## Implications

The study implications address the PPC field at the research, policy, education and practice levels, particularly in a limited-resource setting. Replication of the study in different countries and using longitudinal or experimental designs and different languages would allow further analysis, serving as data comparison and cross-cultural validation of the adapted tool. On the policy level, our findings call for developing a national strategy to structure the implementation of PPC in childhood cancer and other serious conditions while involving parents as indispensable stakeholders and providing them with support in the form of legislation.

On the practice level, the study highlighted the robust contribution of parents in PPC delivery and reaffirms their pivotal contributions of parents’ in the care management process within the paediatric context. Particularly in childhood cancers, enhancing and praising parents’ involvement in care would strengthen their role as experts in their child’s care and potentially promote improved patient outcomes. The success of the partnership in care relies on the parents’ training on PPC skills and bridging the hospital and home setting through outreach programs. In the presence of appropriate internet connectivity, telemedicine may strongly support care delivery at home. Such initiatives would enhance accessibility and efficiency in PPC provision in LMICs.

In the domain of education, our study data constitute an educational needs assessment informing the design of formal and informal educational activities for parents of children with cancer. Recently, Benini *et al* [39] affirmed that ‘parents and other family members should be trained and supported 24/7 in caring for their child at home whenever possible’ (p.e536). Training workshops may promote the parents’ theoretical understanding of PPC and enhance their technical skills in performing PPC tasks.

## Conclusion

Examining the tasks that parents perform in PPC for children with cancer promotes a better understanding of PPC provision within a resource-limited setting. The study findings form the basis for future improvement strategies, which mediate the enhancement of palliative care provision of children with cancer. Despite its limitations, the study brings to light an underexplored perspective for studying PPC and fills a gap in the literature regarding parents’ roles in such care. Hence, the study findings provide evidence regarding the need to optimize this role in PPC and on the potential factors to consider when designing improvement strategies. Above all, the study paves the way toward impactful improvement in research, policy, education and practice of PPC by honouring the parents’ contribution in the care trajectory of children with cancer.

## Conflicts of interest

The authors declare that there is no conflict of interest. The study was conducted in partial fulfilment of the requirements for the degree of Doctor of Philosophy of the Rafic Hariri School of Nursing at the American University of Beirut.

## Funding

This research received no specific grant from any funding agency in the public, commercial or not-for-profit sectors.

## Author contributions

RSR designed the study, executed data collection and analysis and wrote the manuscript. HAH, SN, ELS, JW, SF and MRA supervised the study design and conduct, validated the study results and analysis and reviewed the manuscript.

## Figures and Tables

**Table 1.** Demographic characteristics of the sample (*n* = 105).

Characteristic	Number	Proportion (%)
Gender		
Female	89	85
Male	16	15
Relationship to the child		
Biological parent	103	98
Other (Aunt, Sister)	2	2
Age (years) ^a^		
<30	16	15

30–39	45	43
40–49	37	35
>50	7	7
Marital status		
Married	95	90
Separated/Divorced	7	7
Widowed	2	2
Single	1	1
Nationality		
Lebanese^ b^	94	90
Non-Lebanese (Syrian/Iraqi/Palestinian)	11	10
Highest education level		
Below grade school	21	20
Grade school	25	24
High school	17	16
University	31	30
Graduate school	11	10
Area of residence		
Urban	49	47
Rural	56	53
Religion		
Christian	15	14
Muslim	85	81
Druze	5	5
Employment status		
Employed	23	22
Homemaker	67	64
Unemployed	4	4
Other (student/retired/freelancer)	11	10
Monthly income		
Doesn’t meet basic needs	63	60
Meets basic needs	38	36
Exceeds basic needs	4	4

Table 1 presents the frequency and proportion of each characteristic in the sample

^a^ Participants were, on average, 37.6 years old (*SD* = 7.6).^ b^ Two participants hold other nationalities (Armenian and Syrian) in addition to the Lebanese nationality.

**Table 2.** Symptom prevalence and symptom scores (*n* = 105).

Symptom in the previous week	Prevalence		Symptom score	
	*n*	%	*M*	*SD*
Feeling irritable	64	61	2.1	0.7
Lack of appetite	49	47	1.7	0.6
Nausea	47	45	1.8	0.6
Pain	44	42	2.0	0.6
Worrying	41	39	1.9	0.6
Feeling sad	40	38	1.8	0.6
Lack of energy	36	34	1.8	0.6

Cough	36	34	1.2	0.5
Feeling nervous	34	32	2.0	0.6
Difficulty sleeping	18	17	2.0	0.7

Table 2 illustrates the frequency of symptoms and the mean scores and standard deviation for each symptom. The symptom score is the composite of means of the three symptom dimensions (frequency, severity and distress). The total MSAS score represents the average of symptoms scores. A higher score indicates a more intense symptom experience [26]. The total MSAS score = 1.8 ± 0.45. Global Distress Index = 1.9 ± 0.4.

**Table 3.** PPC tasks and intentions (*n* = 105).

PPC task	Prevalence		Intentions	
	*n*	%	*M*	*SD*
Meeting the emotional needs of my ill child	105	100	–	–
Maintaining my child’s comfort	105	100	–	–
Obtaining necessary equipment and medications	105	100	–	–

Giving medications by mouth	104	99	2.00	–
Attending medical appointments	101	96	2.50	1.73
Reminding my child about medical precautions	101	96	2.75	1.50
Communicating about the child’s illness	95	90	2.60	0.84
Planning activities for the ill child	92	88	3.00	1.08
Meeting the emotional needs of other children in my family	91	87	2.67	0.57
Additional household tasks	91	87	3.21	1.36
Praying with my child	88	84	2.77	1.01
Meeting the emotional needs of my extended family	87	83	2.39	0.85
Obtaining childcare for my ill child	87	83	2.00	0.82
Meeting my own emotional needs	86	82	2.95	1.03
Telling medical information to my child	82	78	2.31	0.63
Managing the side effects of treatment	79	75	2.46	0.76
Meeting the emotional needs of my spouse	79	75	3.12	0.93
Planning activities for the family	78	74	3.00	0.98
Sharing my experience with similar parents	78	74	2.33	0.96
Obtaining childcare for the siblings	78	74	1.60	0.55
Disciplining the ill child	76	72	2.00	0.67
Managing ﬁnances	75	71	2.40	1.07
Following up with the treatment team (e.g., phone calls)	68	65	2.70	0.91
Managing painful events	54	51	2.02	0.51
Getting information about the child’s illness	52	50	2.36	0.98
Managing other childhood illnesses	46	44	2.02	0.39
Managing unexpected events	42	40	2.54	0.80
Taking decisions related to my child’s care	32	30	2.19	0.79
Managing medical devices such as feeding pump	16	15	1.86	0.38
Getting more information about PPC	9	9	3.97	0.81
Discussing PPC with my child’s healthcare team	3	3	3.55	0.89

Table 3 presents the frequency and proportion of PPC tasks performed by the participants during the previous week. The ‘Intentions’ column illustrates the mean and standard deviation of the participants’ likelihood to engage during coming week in PPC tasks that were not performed in the pervious week.
